# Photocured Poly(Mannitol Sebacate) with Functional Methacrylic Monomer: Analysis of Physical, Chemical, and Biological Properties

**DOI:** 10.3390/polym15061561

**Published:** 2023-03-21

**Authors:** Víctor Hevilla, Águeda Sonseca, Coro Echeverría, Alexandra Muñoz-Bonilla, Marta Fernández-García

**Affiliations:** 1 Instituto de Ciencia y Tecnología de Polímeros (ICTP-CSIC), C/Juan de la Cierva, 3, 28006 Madrid, Spain; 2 Interdisciplinary Platform for “Sustainable Plastics towards a Circular Economy” (SUSPLAST-CSIC), 28006 Madrid, Spain; 3 Instituto de Tecnología de Materiales, Universitat Politècnica de València, Camino de Vera, s/n, 46022 Valencia, Spain

**Keywords:** poly(mannitol sebacate), crosslinking, cationic moieties, antimicrobial, toxicity

## Abstract

In this work, we described the formation of polymeric networks with potential antimicrobial character based on an acrylate oligomer, poly(mannitol sebacate) (PMS), and an enzymatically synthesized methacrylic monomer with thiazole groups (MTA). Networks with different content of MTA were prepared, and further physico-chemically characterized by microhardness, water contact angle measurements, and differential scanning calorimetry. Monomer incorporation into the networks and subsequent quaternization to provide thiazolium moieties affected the mechanical behavior and the surface wettability of the networks. Moreover, the introduction of permanent cationic charges in the network surface could give antimicrobial activity to them. Therefore, the antibacterial behavior and the hemotoxicity were analyzed against Gram-positive and Gram-negative bacteria and red blood cells, respectively.

## 1. Introduction

UV curing technology has found wide application in industry, such as in the production of adhesives, decorative or protective coatings, and ultra-fast drying of inks and varnishes. Mostly, its use has been based on acrylic derivatives; however, the increase of biocompatible monomers and initiators has spread their possibilities [1]. The manufacture of biodegradable polymer systems by photopolymerization processes, such as additive manufacturing or 3D printing [2,3,4], has aroused great interest due to their potential and versatility in biomedical applications, e.g., dental restoration or tissue engineering scaffolds [5,6,7,8]. This is due to the fact that the polymeric systems obtained present mechanical properties similar to soft tissues [9] and can be obtained with short and controllable reaction times, at room or physiological temperature, and in the presence of oxygen and/or water, which makes the process very suitable to be carried out in situ.

One of the most promising families of soft polyesters is the poly(polyol sebacate) (PPSe) family, as they have already shown potential biomedical applications [10,11,12,13,14,15]. Poly(glycerol sebacate) (PGSe) is the most studied member of the PPSe family and has been used in various soft tissue engineering applications or as a carrier for anti-cancer drugs [10,11,12,16,17,18,19,20,21,22,23,24,25]. Most of these polymers are commonly easily produced through melt polycondensation reactions instead of enzymatic polymerization. However, high temperatures (typically > 110 °C) and vacuum are required to obtain PGSe networks. Therefore, there is a constant investigation into PGSe functionalization with chemical groups to convert them into photocurable systems and the subsequent production of complex 3D morphologies with mechanical properties matching the mechanical properties of soft tissue. Our group has developed 3D light-cured systems that are based on a poly(glycerol adipate) macromer and a methacrylic monomer that contains thiazole groups, whose quaternization introduces antimicrobial character to the network [26]. Over the years our group has developed antibacterial petroleum-based and biobased polymers bearing cationic azolium groups derived from the vitamin thiamine (B1). These polymers demonstrate excellent antibacterial activity against Gram-positive bacteria, some effect against Gram-negative bacteria and fungus, and negligible toxicity to human red blood cells (RBCs) [27,28,29,30,31,32,33].

In this article, a previously synthesized macromer of acrylated poly(mannitol sebacate) (PMS-Ac) [34] and enzymatically synthesized 2-(4-methylthiazol-5-yl)ethyl methacrylate (MTA) [26] were used to obtain different 3D networks by photopolymerization. These networks were modified by alkylation to introduce permanent cationic charges, with the intention of providing antimicrobial character. The networks’ structural characterization was carried out by infrared spectroscopy; their surface was analyzed by scanning electron microscopy, water contact angle, and surface charge measurements; their thermal properties by differential scanning calorimetry; and their mechanical behavior by microhardness testing. The antimicrobial activity was tested against Gram-positive and Gram-negative bacteria by dynamic contact killing. Finally, the toxicity of these networks was tested against RBCs.

## 2. Materials and Methods

### 2.1. Materials

Sebacic acid (SA, HOOC-(CH_2_)_8_-COOH), mannitol (MA, C_6_H_14_O_6_), triethylamine (TEA, N(CH_3_)_3_), acryloyl chloride, hydrochloric acid (HCl), anhydrous *N*,*N*-dimethylformamide (DMF), deuterated dimethyl sulfoxide (DMSO-d_6_), lithium bromide (LiBr), 5-(2-hydroxyethyl)-4-methylthiazole (Tz, 98%), phosphate buffered saline powder (PBS, pH 7.4), fluorescein sodium salt powder, and cetyltrimethylammonium chloride (CTAC) were acquired from Sigma Aldrich and used as received. Vinyl methacrylate (VMA, TCI Chemicals, 98.0%), iodomethane (MeI, 99.5%, Merck), ethyl acetate (99.8%, Scharlau), n-hexane (96% Scharlau), and 2-benzyl-2-dimethylamino-1-(4-morpholinophenyl)-butanone-1 (Irgacure 369, Ig369, Ciba Specialty Chemicals) were used as they were received. For the antimicrobial test, Columbia agar (5% sheep blood) plates were obtained from Fisher Scientific. Bacteria strains of American Type Culture Collection (ATCC) *Staphylococcus aureus* (*S. aureus*, ATCC 29213), methicillin resistant *Staphylococcus aureus* (MRSA, ATCC 43300), *Escherichia coli* (*E. coli*, ATCC 25922), and *Pseudomona aeruginosa* (*P. aeruginosa*, ATCC 27853) were purchased from Oxoid.

The enzymatic synthesis of MTA was recently reported by our group using Tz and VMA [27]. ^1^H NMR (400 MHz, CDCl_3_, ppm): δ = 8.52 (s, 1H; =CH thiazole), 6.02 (m, 1H; =CH), 5.49 (m, 1H; =CH), 4.22 (t, J = 6.5 Hz, 2H; OCH_2_), 3.06 (t, J = 6.5 Hz, 2H; CH_2_), 2.33 (s, 3H; CH_3_ thiazole), 1.84 (dd, J = 1.2, 1.5 Hz, 3H; CH_3_); ^13^C NMR (400 MHz, CDCl_3_, ppm): δ = 166.9 (C=O), 149.9 and 149.7 (NCs), 135.9 (–C=CH_2_), 126.8, 125.8 (=CH_2_s and C=C–S), 64.1, 25.7, 18.3, 14.7. Poly(mannitol sebacate) (PMS) (Mn and Ð obtained by SEC were 2480 g/mol and 1.22, respectively) and PMS-Ac (~80% of functionalization) were also synthesized according to previously published methods [34]. Briefly, PMS was synthesized from SA and D-mannitol in bulk at 150 °C in a molar ratio SA/MA of 1/1.1. The acrylation was performed with acryloyl chloride in DMF solution. The composition was determined by NMR spectroscopy and the acrylation degree by comparing the signal intensity of the methylene groups on the SA backbone (1.3 ppm) with the signal intensity of the acrylate groups (6.0–6.5 ppm).

### 2.2. Photo-Crosslinking Process

PMS-Ac and MTA were dissolved in acetone (25:75, mixture:acetone, *w*/*w*) in different weight percentages (95:5, 90:10, and 85:15, PMS-Ac:MTA) and using 3.0 wt.% of Ig369 photoinitiator with respect to the total weight of the mixture. They were protected from light and stirred at room temperature until the mixture was homogeneous. Crosslinked samples were prepared on round glass plates (diameter of 12 mm) adding 50 μL of mixture and dried for twenty minutes in a gas hood, allowing the solvent to evaporate. Subsequently, they were exposed to an UV lamp using a UVPTM CL–1000 short-wave photocrosslinker (λ = 313 nm) for 10 min. The photocrosslinked systems were named as nPMS and nPMSx, x being the MTA wt.%.

### 2.3. Quaternization of Crosslinked Samples

Cured samples were repeatedly washed with MeOH and then quaternized with 1.0 mL of MeOH and 10 μL of MeI in the absence of light at 40 °C. After time, samples were extracted from the medium, subjected to a new sequence of washes with MeOH, and dried under vacuum until constant weight. The quaternized systems were named as nPMSxQ.

**Scheme 1:**
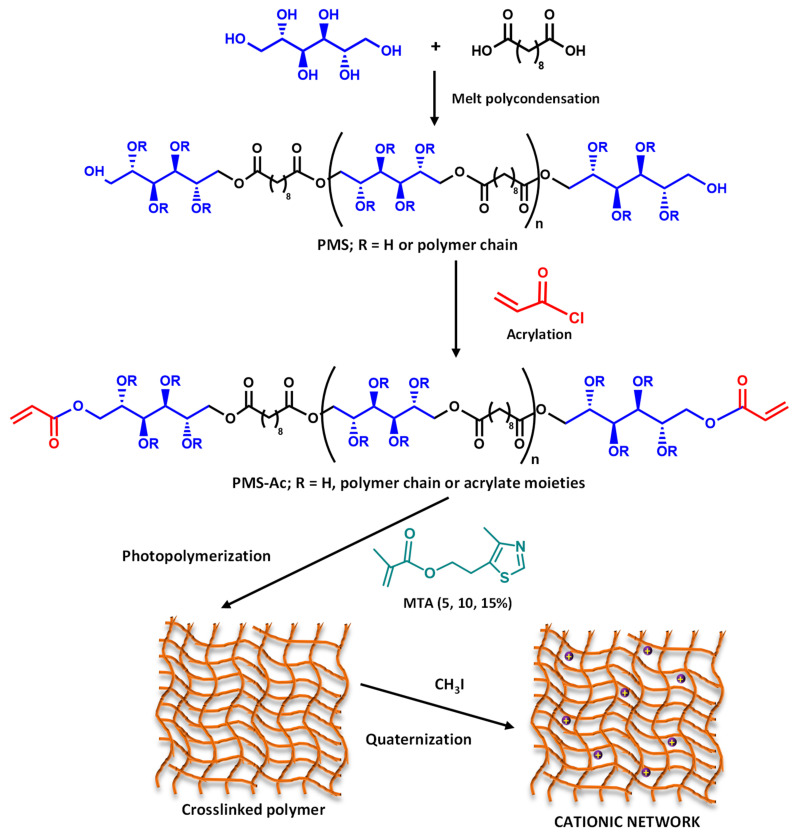
Schematic representation of the preparation process of active crosslinked polymers.

Scheme 1 presents the synthesis of PMS and its functionalization, followed by the addition of MTA and its photocuring with UV light, and the subsequent quaternization process of the networks obtained.

### 2.4. Characterization 

NMR Spectroscopy. ^1^H NMR spectra were recorded on a Bruker Avance III HD-400AVIII spectrometer at room temperature using DMSO-d_6_ and CDCl_3_ as internal references for reported chemical shifts. 

Size exclusion chromatography (SEC). Relative molecular weights of PMS and PMS-Ac were determined by SEC using a Waters Division Millipore system. This system was equipped with a Waters 2414 refractive-index detector and the used eluent was N,N-dimethylformamide (Scharlau, 99.9%) containing 0.1% of LiBr, with a flow rate of 1 mL/min at 50 °C. Poly(methyl methacrylate) standards (Polymer Laboratories Ltd., Church Stretton, UK) were used to calibrate the system ranging from 1.4 × 10^6^ and 5.5 × 10^2^ g/mol. 

Scanning electron microscopy (SEM). SEM images of the surface of the networks were taken using a Philips XL30 (The Netherlands) with an acceleration voltage of 25 kV. The networks were coated with gold prior to scanning.

Contact angle. The static water contact angle (WCA) of the samples was determined on the surface of the prepared films using a KSV Theta goniometer (KSV Instruments Ltd., Espoo, Finland) in contact mode at 25 °C. The contact angle was measured at least eight times on different sites of the surface using a drop volume of 3.0 μL. Each value reported is the average of eight measurements ± SD (standard deviation).

Surface charge. This was measured following a method previously described in the literature [35]. Film samples were placed in 1.0 mL of 1 wt.% aqueous sodium fluorescein solution for 10 min. After that, samples were extensively washed with distilled water. Then, the fluorescein was desorbed from the sample by treating the film with 1.0 mL of 0.1 wt.% CTAC solution for 10 min with shaking at 200 rpm. This was repeated at least two times. Subsequently, the amount of fluorescein obtained in the extraction was determined at λ = 501 nm measured by UV-vis spectroscopy (Lamda 35, PerkinElmer Spain S.L., Madrid, Spain) in a solution prepared by adding 0.3 mL of 100 mM phosphate buffer (pH 7.4). The concentration of fluorescein was then calculated using an extinction coefficient [36] of 77 M^–1^ cm^–1^ assuming a relationship of 1:1 for fluorescein to each accessible cationic thiazolium group.

Differential scanning calorimetry (DSC). Thermal data were obtained by DSC using a TA Instruments Q2000 series equipped with a refrigerated cooling system. Runs were conducted with a sample weight of ~2 mg under a flow of dry nitrogen of 50 cm^3^/min. Samples were equilibrated at −60 °C and heated at 10 °C/min up to 200 °C, followed by quickly cooling to −60 °C, and then heated again to 200 °C at the same rate. 

Microhardness. Microindentation measurements were performed using a Vickers indentor attached to a Leitz microhardness (MH) tester. A contact load of 0.96 N for a time of 25 s was employed. MH values in MPa were calculated according to the following relationship [37]:

$$\mathrm{MH}=2\;\sin\;68{}^{\circ}\;\left(\frac{P}{d^{2}}\right)$$

where P is the contact load and d is the diagonal length of the projected indentation area (in N and mm, respectively). Diagonals were measured in the reflected light mode within 30 s of load removal, using a digital eyepiece equipped with a Leitz computer-counter-printer. Each value reported is the average of several measurements.

X-Ray diffraction. A Bruker D8 Advance X-ray diffractometer (Bruker Scientific LLC, Billerica, MA, USA) equipped with a CuKα (λ = 0.154 nm) source was employed to obtain an X-ray diffraction pattern. The sample was framed on an appropriate holder and scanned between 10° to 40° (2θ) with a scanning step of 0.05°, a collection time of 18 s per step, and 40 kV of operating voltage.

Antibacterial Assays. The antibacterial activity of the different UV-cured PMSxQ networks was measured following the E2149-20 standard method from the American Society for Testing and Material (ASTM) [38] against E. coli and S. aureus bacteria. Bacterial cells were grown on 5% sheep blood Columbia agar plates for 24 h at 37 °C. After that, the bacterial suspensions were prepared in saline medium using ca. 0.5 McFarland turbidity scale and further diluted to ca. 10^6^ colony-forming units (CFU)/mL with PBS. Next, each UV-cured sample was placed in sterile falcon tubes, containing 1.0 mL of the tested inoculum and 9.0 mL of PBS, to reach a working solution of ca. 10^5^ CFU/mL. Tubes in the absence of networks and with nPMS and nPMSx films were used as blank control experiments. After 24 h of contact, 10-fold serial dilutions were performed, and each was spread onto 5% sheep blood Columbia agar plates. Finally, the colonies were counted, and the reduction percentage was calculated. The measurements were performed in triplicate.

Hemotoxicity analysis. RBCs were collected in heparinized tubes from healthy donors from the Hospital Universitario de Móstoles and were centrifuged at 3100 rpm for 10 min and washed three times with cold PBS to remove plasma and white blood cells. The solution was suspended to 5% (*v*/*v)* in PBS to yield the RBC stock suspension and used immediately. Each network (4.3 ± 0.5 mg) was added to a tube with 4 mL of RBC solution. Positive control (100% hemolysis) was checked by adding 1 mL of Triton X-100 solution, and a negative control (0% hemolysis) was performed by adding 3 mL of RBC stock in 1 mL of PBS. The tubes were incubated at 37 °C for 1 h and 24 h and then centrifuged at 3100 rpm for 10 min to settle the non-lyzed cells. Then, 150 μL of supernatant from each tube was added into a sterile 96-well microplate and the absorbance of wells was measured at 550 nm. The percentage of hemolysis was determined as follows:

Hemolysis (%) = (A − A_0_)/(A_100_ − A_0_) × 100 (1)

where A is the absorbance of the analyzed network, A_0_ the absorbance of the negative control (0% hemolysis), and A_100_ the absorbance of the positive control (100% hemolysis). Each percentage is given as the average and standard errors from different experiments performed in triplicate.

Statistical analysis. The analysis of variance (ANOVA) was performed to detect any significant differences between the factors at *p* < 0.05. The software used was Origin 8.5 (Northampton, MA, USA).

## 3. Results

**Figure 1:**
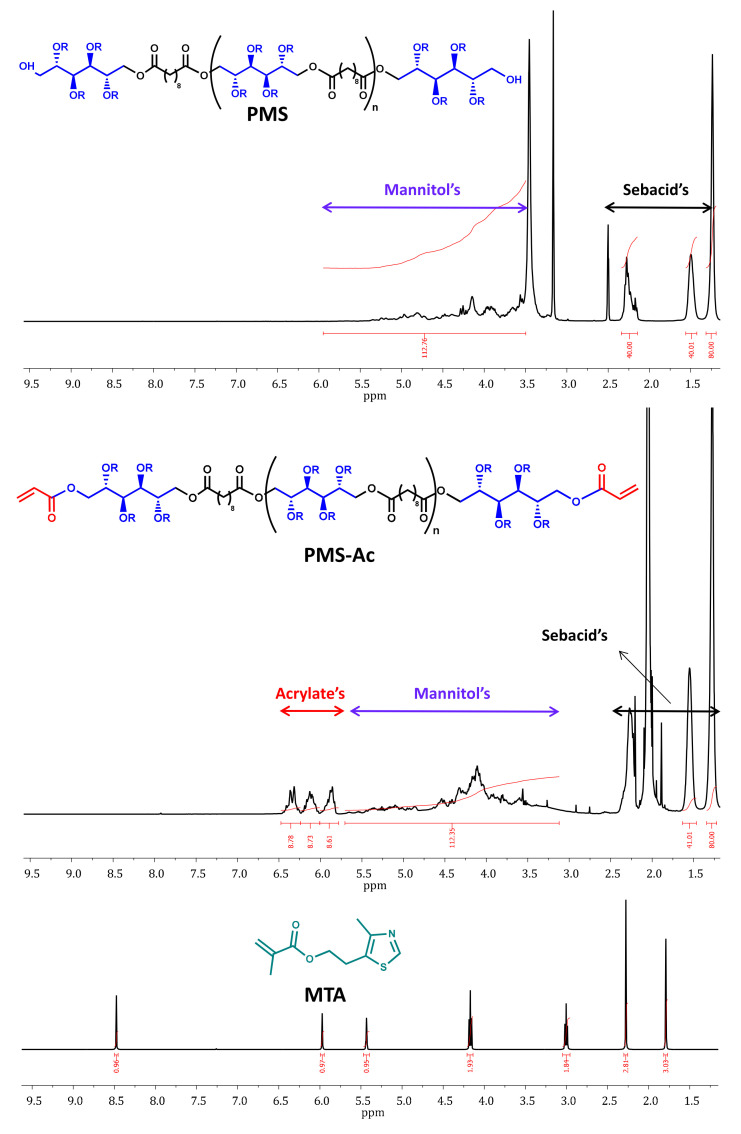
^1^H NMR spectra of PMS, PMS-Ac, and MTA monomer.

The synthesis of a PMS polyester from D-mannitol and sebacic acid and its subsequent functionalization with acrylate groups was successfully performed previously [34]. These polymers were incorporated into poly(lactic acid) to create functionalized fibers. The subsequent attachment of a thiol functional dye to the surface was then a possible modification. In this work, the acrylate groups will be used for a crosslinking reaction. Figure 1 displays the ^1^H NMR spectra of PMS and PMS-Ac as well as the MTA monomer. The PMS macromer presents almost the same ratio of monomers than those initially used, in this case a SA/MA value of ~1/1.4. The degree of acrylation is 87 ± 1%, which makes it a valuable material to produce 3D networks. 

In this work, photocrosslinking of PMS-Ac in the presence of Ig369 photoinitiator (to give nPMS), and copolymerization with the MTA functional monomer (to give nPMSx) were successfully performed. After crosslinking, the separation of the different networks from the glass dishes was easily done by immersing them in methanol.

As the main aim of this work was to obtain active systems with potential applications in biomedicine, the resulting networks functionalized with thiazole groups were quaternized by an N-alkylation reaction to provided thiazolium moieties with a permanent cationic charge and thus impart antimicrobial activity [30,39]. It is important to note that the quaternized MTA monomer provokes incompatibility with PMS-Ac and, therefore, an unsuccessful crosslinking. For this reason, the N-alkylation process was carried out after network formation.

**Figure 2:**
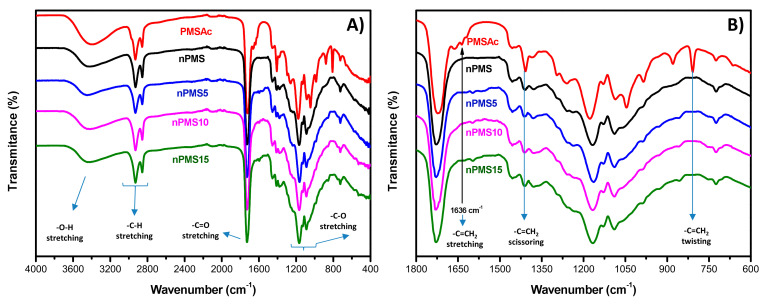
(**A**) ATR–FTIR spectra of all non-quaternized networks, and (**B**) magnified view of the 1800–600 cm^−1^ region.

Figure 2 shows the FTIR spectra of different networks prior to the quaternization process. In these spectra, the disappearance of the C=CH_2_ stretching vibration at 1636 cm^−1^ can be observed, confirming that the crosslinking has been successful in all cases. Characteristic bands of both network components can also be observed, such as a stretching vibration of hydroxy groups around 3470 cm^−1^, of methylene groups at 2924 cm^−1^ and 2851 cm^−1^, and of C=O and C–O at 1740 cm^−1^ and 1164 cm^−1^, respectively. However, there is not much difference between the spectra of the different networks. To ratify that no soluble part is removed during the quaternization process, the networks were exposed to an extraction process by immersion in methanol for one week. After this procedure, no solid residue was found, indicating that there were no free polymer chains.

**Figure 3:**
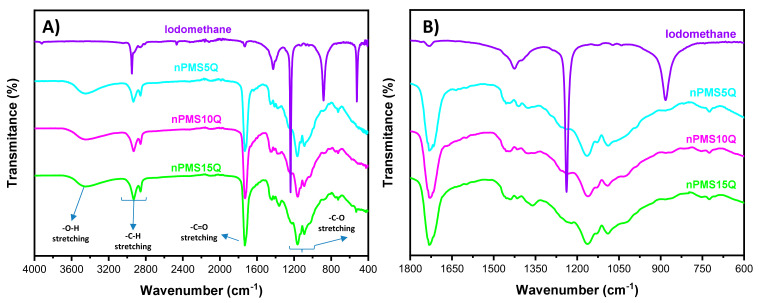
(**A**) ATR–FTIR spectra of all quaternized networks, and (**B**) magnified view of the 1800–600 cm^−1^ region.

Afterwards, alkylation of the crosslinked polymers was performed. To confirm that there was no excess alkylation agent, the networks were cleaned with methanol several times and a Lugol test was performed to prove that the samples were totally free of methyl iodide. In addition, Figure 3 shows the ATR-FTIR spectra of all quaternized networks and that of iodomethane. In these spectra, free iodomethane is not observed, and the three network spectra do not show any difference between them, as happens when the networks are not quaternized.

**Figure 4:**
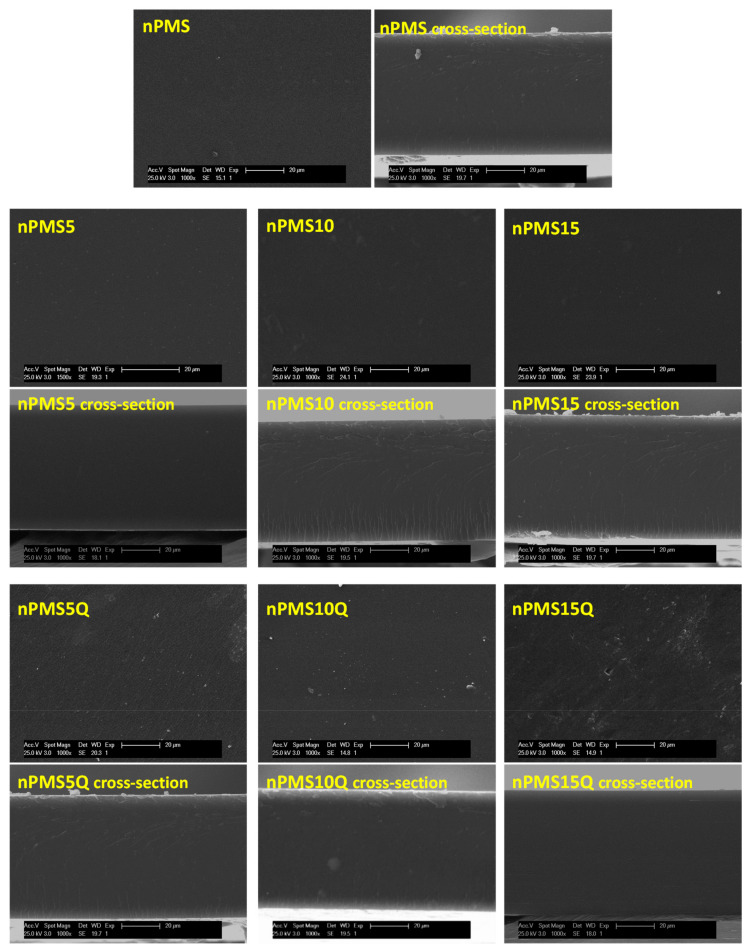
SEM micrographs of nPMS, nPMSxn, and nPMSxQ networks’ surface and the corresponding micrographs of their cross-sections obtained by fracture.

Figure 4 shows that flat surfaces are obtained in the networks before and after quaternization. As can be seen, the treatment does not change the morphology of the material. The important feature that is detected is the highly crosslinked character of the networks (see the cross-sections in Figure 4), which is somehow expected since the degree of PMS methacrylation is very high (~80%). This fact could have an enormous effect on the mechanical properties as well as bioactivity of the networks.

The accessible cationic units on the surface were measured using a method based on the adsorption of fluorescein by cationic units of the films to confirm the modification. Later, adsorbed fluorescein was desorbed with CTAC and quantified by UV-vis spectroscopy. The values obtained by this technique, of cationic groups per square centimeter, are also collected in Table 1 and, as expected, these values increase with an increase in MTA content. The obtained data are similar to those reported in the literature [35,40,41]. Depending on the charge density of cationic substrates, the induction of bacterial death can be decisive [40,42,43]. It is proposed that the removal of divalent counterions from the bacteria during adsorption on charged surfaces induces disruption of the bacterial envelope and non-viability [4,44]. The current values in the order of 10^16^ N^+^/cm^2^ could indicate that the systems have potential to kill bacteria.

The study of the wettability of the samples was carried out by measuring the WCAs (see Table 1). The SEM analysis demonstrated that the networks possess flat surfaces and there are no significant differences between quaternized and non-quaternized samples. As such, the WCA values should be related to the chemical composition at the surface. All values of WCA are lower than 90°, so the surfaces can be considered hydrophilic. The obtained WCA value of nPMS is higher than the literature value for PMS cured at 140 °C using SA monomer, which gives 32.2° ± 9.0° and 40.4° ± 9.3° depending on the ratio between monomers [45]. The use of photocuring instead of thermal crosslinking makes the system more rigid. MTA could be considered a hydrophobic molecule, so its incorporation into the network could also produce an increase in the WCA values. As can be seen in Table 1, the incorporation of MTA causes a slight increment of WCA values, which is significant when the MTA proportion is higher than 5%. However, a higher amount of MTA in the network does not considerably increase the WCA values. Likewise, the quaternization process causes an increase, in a similar trend to that observed in non-quaternized samples. This increase could be due to the repulsive forces between hydroxy groups of mannitol and thiazolium groups as well as steric hindrance.

In Table 1, the results obtained by MH measurements are also gathered. It is important to analyze the effect of the MTA incorporation and its quaternization on the mechanical properties of the crosslinked polymers. MH is proportional to Youngs’ modulus regardless of the material composition [46] and corresponds to resistance against permanent indentation. An acceptable surface hardness plays an important limitation in certain applications, i.e., dental restoration [47]. As can be observed in Table 1, the nPMS network is highly rigid but the incorporation of 5% of MTA comonomer produces a drastic softness in the system of ca. 90 MPa. The increment of MTA raises the MH value; almost reaching the initial value. This can be explained since the network could possess a higher crosslinking density due to a lower viscosity in the mixture. The quaternization process increased the MH, which could be attributed to the formation of new inter and intramolecular interactions between the quaternary nitrogen and the acid groups in the networks, which create more rigid films.

**Figure 5:**
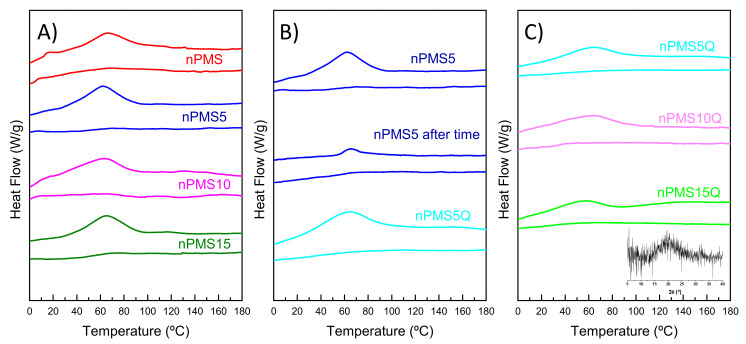
DSC curves of (**A**) all nPMSx and nPMS; (**B**) nPMS5, nPMS5 after time, and nPMS5Q and (**C**) all nPMSxQ networks.

Table 1 also collects the glass transition temperatures (T_g_s) of nPMSx and nPMSxQ networks. It is important to establish that there is no uncured polymer after the photocrosslinking process. Figure 5 shows the curves of first and second heating runs of the nPMSx, nPMSxQ, and PMS networks. All samples display an exothermic transition, which can be attributed to physical aging. After fast cooling and the second heating run the glass transition is easily identified. To confirm that there is no other transition, the nPMS5 was analyzed again after three months of the initial scan (see Figure 5B). 

A small peak due to the physical aging is observed. This peak could be due to polymer crystallization; however, the amorphous halo in the X-ray diffraction of the network (see inset in Figure 5C) indicates the contrary. The incorporation of MTA monomer in the network increases the T_g_ but the change between 5% and15% does not modify significantly its thermal behavior. The alkylation modification also does not provoke changes in T_g_, as can be seen in Figure 5C.

**Figure 6:**
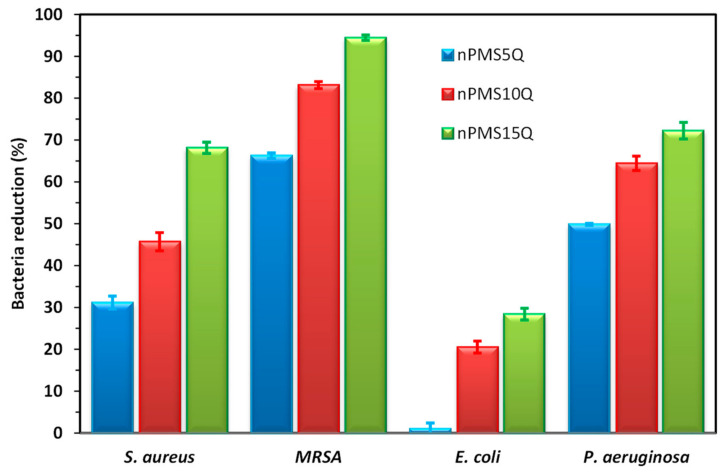
Antibacterial activity expressed as the percentage of bacterial reduction (%) by nPMS5Q, nPMS10Q, and nPMS15Q networks.

As mentioned, the permanent cationic groups within the network could provide antimicrobial character [42,48]. Antibacterial testing was performed against *S. aureus*, methicillin resistant *S. aureus*, *E. coli*, and *P. aeruginosa* strains. Figure 6 shows the bacterial reduction of the tested bacteria against the nPMSxQ systems. The nPMSx networks were also used as a control, since they should not present activity. Essentially, the non-quaternized networks do not present antimicrobial character in contrast to the quaternized networks. Their activity is higher against Gram-positive than against Gram-negative bacteria. This fact is attributed to their proposed mechanism of action, which is electrostatic attraction between the cells and the polymer, disruption of the membrane cell, and finally, cell death [4,44]. In the case of Gram-negative bacteria, their double-layered membrane makes it more difficult to break than for Gram-positive bacteria, which have only one. Although the quaternized samples do not present a strong effect against bacteria, they are able to exhibit some activity which increases as the MTA content increases in the network. The difference in comparison to other systems that contain similar amounts of MTA in their structure, is that the nPMSxQ networks are more hydrophilic, which can hinder their antibacterial activity. 

In addition, the hemotoxicity of these systems was evaluated against RBC for 1 h and 24 h of contact and the results are displayed in Table 2. As can be seen, the quaternized and non-quaternized networks are essentially not hemolytic, with hemolysis percentages much lower than 5%, denoting their non-toxicity. This fact is something expected since the networks are not bactericidal but simply bacteriostatic.

## 4. Conclusions

Networks of modified poly(mannitol sebacate) alone and with 2-(4-methylthiazol-5-yl)ethyl methacrylate in different proportions were obtained. Subsequent methylation of the triazole groups provided a cationic character to the systems. The analysis of their water contact angle indicated their hydrophilic character, which was increased with an increase in the content of monomer that contained triazolium groups. Moreover, the mechanical behavior was strongly affected by the incorporation of monomer in the mixture, while the methylation induced an increase in the stiffness of the networks. A thermal study revealed their amorphous character and the absence of a soluble fraction in the networks. The contact evaluation with different bacteria showed a weak antimicrobial character because the incorporation of 15% of thiazole-containing monomer may not be enough to obtain an effective bactericidal system. The high degree of modification makes the material highly crosslinked and rigid, which could give a negative antimicrobial effect. Despite this, the networks were not hemotoxic. Nevertheless, this study has proved the potential of modified poly(mannitol sebacate), which was able to crosslink alone or with other functional monomers, and expands the biomedical applications of these networks.

## Figures and Tables

**Table 1:** Surface properties of non-quaternized nPMSx and quaternized nPMSxQ networks. The superscript letters represent significant differences with a *p* < 0.05. The glass transition temperature (T_g_) has an error of ±1 °C.

Networks	WCA (°)	Charge × 10^−16^ (N^+^/cm^2^)	MH (MPa)	T_g_ (°C)
nPMS	57.5 ± 2.4 ^a^	-	123.6 ± 7.5 ^a^	36
nPMS5	61.0 ± 2.1 ^a,b^	-	30.5 ± 2.2 ^b^	54
nPMS5Q	63.1 ± 1.6 ^b^	1.0 ^a^	52.6 ± 6.2 ^c^	53
nPMS10	61.4 ± 3.3 ^b^	-	40.6 ± 4.4 ^b^	54
nPMS10Q	73.7 ± 1.8 ^c^	1.7 ^b^	85.3 ± 8.5 ^d^	55
nPMS15	61.8 ± 2.1 ^b^	-	93.9 ± 7.5 ^d^	57
nPMS15Q	76.2 ± 3.4 ^c^	4.8 ^c^	103.1 ± 5.0 ^e^	57

**Table 2:** Hemotoxicity percentage values of nPMSx and nPMSxQ networks against RBC after 1 h and 24 h of contact.

Network	Hemolysis (%) 1 h	Hemolysis (%) 24 h
nPMS5 nPMS5Q	0.5 ± 0.1 0.6 ± 0.5	0.8 ± 0.1 0.8 ± 0.1
nPMS10 nPMS10Q	1.2 ± 0.1 0.2 ± 0.1	0.7 ± 0.1 0.9 ± 0.1
nPMS15 nPMS15Q	0.9 ± 0.1 0.3 ± 0.1	0.5 ± 0.1 0.5 ± 0.1

## Data Availability

The data presented in this study are available on request from the corresponding author.
